# To bind, or not to bind, that is the question…

**DOI:** 10.1107/S2052252522008685

**Published:** 2022-09-01

**Authors:** Wayne F. Anderson

**Affiliations:** a Northwestern University Medical School, 303 E. Chicago Ave, Chicago, IL 60611, USA

**Keywords:** organism-dependent studies, drug interactions, drug transport, human serum albumin, NSAIDs, anti-inflammatory drugs, ketoprofen

## Abstract

The unexpected findings that are described by Czub *et al.* [ *IUCrJ* (2022), **9**, 551–561] provide a very interesting study demonstrating how small differences in structure can result in significant changes in the relative affinities of the seemingly promiscuous binding sites that are seen with serum albumins.

Over many years the examination of protein sequences and structures has led to the conclusion that related sequences indicate related structures and related functions. Due to that observation, biochemists have the expectation that homologous proteins from related organisms will serve as excellent models for each other and behave in essentially identical manners, and therefore that results from one can be transferred to another. The paper by Czub *et al.* (2022 ▸), in this issue of **IUCrJ** , demonstrates that there are limits to these generalizations. The authors of the paper examine the structures of complexes of ketoprofen with human serum albumin and compare the results with complexes of bovine, equine and leporine serum albumins with the same drug (Czub *et al.*, 2020 ▸). The surprising finding is that although these mammalian species are close enough relatives that their serum albumin sequences, as expected, exhibit high similarity to each other, they nonetheless differ in which sites ketoprofen is bound (Fig. 1 ▸). Serum albumins are unusual in that, unlike your typical enzyme, they have evolved to have multiple binding sites for ligands and must be rather promiscuous in the ligands that they bind. The unexpected findings that are described in this paper should be of special interest to people studying protein–ligand interactions.

What may seem, at first glance, to be a rather esoteric and specialized paper has, in fact, a number of important lessons. The presented results demonstrate how small differences in binding site structures and in protein–ligand interactions can result in significant changes in relative affinities at the various sites and a surprising lack of similarity among the homologous proteins. There are also important practical consequences of the promiscuous binding of drugs and drug-like small molecules by serum albumin. A great many drugs are bound to proteins in serum and most of those are bound by serum albumin. Because different compounds may, or may not, compete for the same binding sites on serum albumin, there is the potential for unexpected drug–drug interactions affecting the free drug concentrations and pharmacokinetics.

The many binding sites observed in serum albumins are all characterized by a preference for hydro­phobic ligands, especially ones that are anionic. As a consequence, when the proteins are purified they often carry fatty acids along with them. The characterized fatty acid binding sites frequently overlap with the drug binding sites. This is one of the complicating factors that make defining the ligand binding properties of serum albumins difficult. The detailed interpretation of which sites bind which ligands is not only complicated by differences in the preparation of the serum albumin and thus the fatty acids that are bound, but also the crystallization conditions, the crystal packing contacts, whether the complex was co-crystallized or obtained by soaking, the solvent used for the ligand stock solution and the concentration of ligand that is used. In spite of the large number of possibly confounding factors (that the authors of this paper are well aware of), the important observation remains that the four different mammalian serum albumins clearly diverge in the relative affinities for the *R* and *S* isomers of ketoprofen, and other drugs, at the ten possible drug binding sites on each protein.

Serum albumins have been a favorite of physical biochemists for a very long time. They have been used as model proteins to study binding energetics and multiple equilibria. For example, a look back at Tanford’s classic textbook *Physical Chemistry of Macromolecules* (Tanford, 1961 ▸) demonstrates how important serum albumins were as models of protein behavior and how confusing the results could be. They are also confusing because serum albumins bind varying numbers of a great many different ligands. Knowing the binding constants for different ligands still leaves many uncertainties. A particular difficulty associated with multiple, different, but related sites is that without structural data, binding affinities do not reveal the location of those sites.

The unexpected findings that are described in this paper provide a very interesting study of the seemingly promiscuous binding that one sees with serum albumins. In most studies, one looks at functionally important, evolutionarily conserved ligand–protein interactions and does not see the complexity of multiple sites that is observed with the serum albumins. These, and other, structures of serum albumins with different ligands that are available in the Protein Data Bank (PDB) provide a very interesting system for testing drug discovery software that can be used to design drugs that provide specificity among a family of related sites.

## Figures and Tables

**Figure 1:**
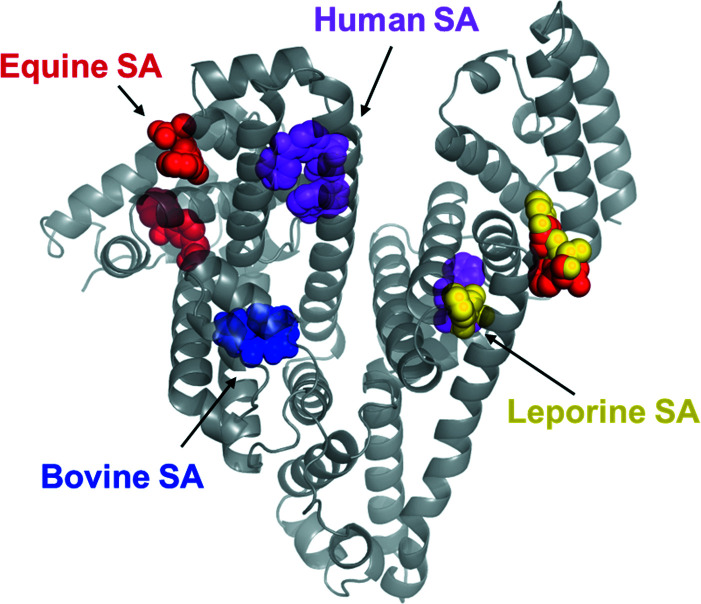
The ketoprofen binding sites from various organisms.
